# Functional Verification of Differentially Expressed Genes Following DENV2 Infection in *Aedes aegypti*

**DOI:** 10.3390/v17010067

**Published:** 2025-01-06

**Authors:** Xiaoli Chen, Xinyu Zhou, Xiaoxue Xie, Bo Li, Teng Zhao, Haotian Yu, Dan Xing, Jiahong Wu, Chunxiao Li

**Affiliations:** 1 The Key Laboratory of Environmental Pollution Monitoring and Disease Control, School of Public Health, Ministry of Education, Guizhou Medical University, Guiyang 550025, China; 2 State Key Laboratory of Pathogen and Biosecurity, Beijing 100071, Chinayuhaotianr@163.com (H.Y.);; 3 The Key and Characteristic Laboratory of Modern Pathogen Biology, College of Basic Medicine, Guizhou Medical University, Guiyang 550025, China

**Keywords:** *Aedes aegypti*, Aag2 cells, DENV2, overexpression, siRNA

## Abstract

The dengue virus (DENV) is primarily transmitted by *Aedes aegypti*. Investigating genes associated with mosquito susceptibility to DENV2 offers a theoretical foundation for targeted interventions to regulate or block viral replication and transmission within mosquitoes. Based on the transcriptomic analyses of the midgut and salivary glands from *Aedes aegypti* infected with DENV2, alongside analyses of Aag2 cell infections, 24 genes potentially related to the regulation of *Aedes aegypti* infection with DENV2 were selected. By establishing transient transfection and overexpression models of *Aedes aegypti* Aag2 cells, and mosquito target gene interference models, the difference in viral load before and after treatment was compared, and the effects of DEGs on viral replication were evaluated. After overexpressing 24 DEGs in Aag2 cells, 19 DEGs showed a significant difference in DENV2 RNA copies in the cell supernatant (*p* < 0.05). In adult mosquitoes, knocking down defensin-A, defensin-A-like, and SMCT1 respectively reduced the DENV2 RNA copies, while knocking down UGT2B1 and ND4 respectively increased the DENV2 RNA copies. In this study, to assess the role of genes related to DENV2 replication, and transient transfection and overexpression models in Aag2 cells and mosquito gene knockdown models were established, and five genes, defensin-A, defensin-A-like, SMCT1, UGT2B1, and ND4, were found to have an impact on the replication of DENV2, providing a reference basis for studying the complex mechanism of mosquito–virus interactions.

## 1. Introduction

The dengue virus (DENV) is a single-stranded, positive-sense RNA virus, which can be classified into four serotypes based on antigenic differences, with DENV2 being more prevalent and dangerous [1]. DENV causes a range of diseases, including dengue fever (DF), dengue hemorrhagic fever (DHF), and dengue shock syndrome (DSS) [2]. As one of the most significant mosquito-borne infectious diseases globally, DF predominantly affects tropical and subtropical regions, causing millions of infections annually and placing nearly half of the global population at risk due to its increasing incidence and infection rates [3,4]. DENV is primarily transmitted by *Aedes aegypti.* While vector-borne viruses often result in severe pathological symptoms in humans, they typically cause no or only mild pathological effects in mosquito vectors [5,6]. Nevertheless, mosquitoes infected with arboviruses activate innate immune responses, altering gene expression profiles and driving complex host–pathogen interactions.

After viral infection, mosquitoes modulate their basic physiological processes, particularly through innate immune responses and defense mechanisms [7,8,9]. These immune regulatory pathways primarily include RNA interference (RNAi), Toll, and JAK-STAT signaling [10,11,12]. Additional mechanisms involve microRNAs (miRNAs), type I lectin responses, the complement system, the PI3K/Akt pathway, autophagy, and apoptosis [13,14]. Together, these processes are essential for the replication and transmission of viruses within their natural life cycle. However, the key factors and regulatory mechanisms underlying mosquito infection remain poorly understood. Investigating genes involved in mosquito–virus interactions may offer valuable insights into how mosquitoes serve as highly efficient vectors for dengue fever. Moreover, such studies may uncover target genes capable of disrupting viral replication and transmission.

To investigate gene functions involved in mosquito–virus interactions, cell lines derived from mosquito hosts provide a valuable and simplified platform for studying insect biology and virology [15]. The Aag2 cell line, derived from *Aedes aegypti* embryos, has been shown to exhibit immune activity, with induced responses closely resembling those observed in individual *Aedes aegypti* mosquitoes [15,16,17,18,19,20]. Furthermore, the Aag2 cell line supports continuous viral replication, making it an ideal model for a variety of pathogen-related in vitro experiments [19,20,21,22,23,24]. Small interfering RNAs (siRNAs), which are double-stranded RNA molecules 20–25 nucleotides in length, play a critical role in gene silencing. Upon entering the cytoplasm of target cells, siRNAs form an RNA-induced silencing complex that degrades and silences mRNA, thereby suppressing target gene expression [25]. Techniques such as the construction of gene expression vectors, transfection methods, and RNA interference (RNAi) technology provide powerful tools for functional gene studies and target gene screening [26,27,28,29].

In this study, we analyzed the transcriptome sequencing data of the midgut and salivary glands of *Aedes aegypti* infected with DENV2 from the NCBI Sequence Read Archive (SRA) database (BioProject ID: PRJNA1197782) [30], as well as the transcriptome data of Aag2 cells infected with DENV2 [31], 24 genes potentially related to the regulation of *Aedes aegypti* infection with DENV2 were selected. By constructing transient transfection and overexpression models in Aag2 cells, along with gene knockdown models in mosquitoes, viral loads were compared before and after gene overexpression in Aag2 cells and gene knockdown in mosquitoes. This approach facilitated the screening and verification of the target gene’s replicative role in vector–virus interactions. These findings provide a theoretical foundation for the targeted regulation of mosquito-borne virus infections and transmission.

## 2. Materials and Methods

### 2.1. Virus, Cells, Plasmids and Mosquitoes

The DENV2 Guangdong strain was obtained from the Guangdong Provincial Centers for Disease Control and Prevention, China [32]. *Aedes aegypti* Aag2 cells were cultured in Schneider’s Drosophila Medium (SDM, Gibco, Waltham, MA, USA) supplemented with 10% fetal bovine serum (FBS, Gibco, Waltham, MA, USA), and incubated at 28 °C with 5% CO_2_ in a cell culture incubator. C6/36 cells were cultured in RPMI 1640 Medium (RPMI 1640, Gibco, Penrose, NZ) supplemented with 10% FBS and incubated at 27 °C with 5% CO_2_ in a cell culture incubator.

**Figure 1:**
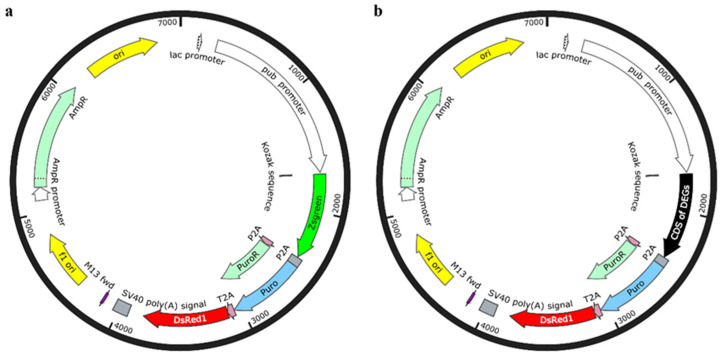
Plasmid vectors: (**a**) Control plasmid vector; (**b**) Experimental plasmid vector. In the plasmid vectors, the Pub promoter refers to the polyubiquitin promoter sequence from *Aedes aegypti*, which significantly enhances the long-term stability of gene expression. ZsGreen represents Enhanced Green Fluorescent Protein (EGFP), while DsRed indicates Red Fluorescent Protein. The CDS of DEGs represents the protein-coding sequences of the 24 selected differentially expressed genes (Table 1).

PSL1180polyUBdsRED was a gift from Leslie Vosshall (Addgene plasmid #49327; http://n2t.net/addgene:49327, accessed on 10 December 2024; RRID: Addgene_49327). Experimental and control plasmids were designed using SnapGene software, employing the double enzyme digestion method to avoid random cutting and reverse ligation. EGFP and puromycin sites were incorporated into the original vector, with EGFP serving as a control for the overexpression experimental vector (Figure 1a). The DsRed reporter gene was used to indicate the expression and localization of the target gene in cells. In the experimental plasmid, the EGFP sequence was replaced with the coding region of the gene of interest (Figure 1b). After designing the vector, Sangon Bio (Shanghai, China) Co., Ltd. was commissioned to synthesize it.

The mosquitoes used in this study were *Aedes aegypti* of the Hainan Haikou strain, which were reared at the State Key Laboratory of Pathogen and Biosecurity. The rearing conditions were as follows: temperature 27 ± 1 °C, relative humidity 75 ± 5%, and a photoperiod of 14 h light and 10 h dark. After eclosion, adult female mosquitoes were fed a sucrose solution with concentrations ranging from 8% to 10%.

### 2.2. Differentially Expressed Genes (DEGs) Related to DENV2 Infection and Replication

In previous research, our laboratory conducted an in-depth analysis of the transcriptome data of Aag2 cells [31], as well as the midgut and salivary glands of *Aedes aegypti* (BioProject ID: PRJNA1197782) [30], using bioinformatics analysis methods, and identified differentially expressed genes (DEGs). On this basis, this study further selected 24 differentially expressed genes (DEGs) related to DENV infection from the salivary glands, midgut, and Aag2 cells of *Aedes aegypti*, using the criteria of at least one transcriptome with Padj < 0.05 and |Fold Change| > 1. And the DEGs were further investigated using the overexpression model.

### 2.3. Transfection

First, Aag2 cells were subcultured in a 12-well plate (Thermo Fisher Scientific, Waltham, MA, USA) using SDM containing 2% FBS at a density of 1 × 10⁵ cells/mL, with 1 mL/well. The cells were incubated for 3 days before performing the transfection experiments. FuGENE@6 Transfection Reagent (Promega, Madison, WI, USA) was used for transfection with the original PSL1180polyUBdsRED vector. To optimize the transfection system, four different ratios of transfection reagent (μL) to plasmid vector (μg) were tested (4:1, 6:1, 8:1, and 10:1), with the 8:1 ratio being the optimal condition. The optimal transfection system for each well of a 12-well plate was as follows: 1 μg plasmid, 8 μL transfection reagent, and Opti-MEM medium to a final volume of 50 μL. For the transfection procedure, the transfection reagent was added to Opti-MEM medium and incubated at room temperature for 5 min. The plasmid was then added, followed by a 15 min incubation at room temperature. This mixture was then applied to the cells, which were incubated for 24 h. After 24 h, red fluorescence expression was observed under a fluorescence microscope, and DENV2 infection was subsequently performed.

### 2.4. Preparation of DENV2 Suspension Using C6/36 Cells

C6/36 cells were cultured in 1640 medium supplemented with 10% FBS. When the cell density reaches 80–90% and the cells are in good condition, preparation of the DENV2 suspension can proceed. After removing the original medium from the T75 cell culture flask and retaining a small amount of liquid, add 1 mL of DENV2 suspension to each cell flask for mixing. Gently shake the culture flask to ensure the DENV2 suspension fully contacts the C6/36 cells. After thorough mixing, immediately place the culture flask in a 27 °C, 5% CO_2_ cell incubator. Shake it every 15 min and allow it to adsorb for 1 h. After adsorption is complete, add 12 mL of 1640 medium containing 2% FBS along the side wall of the cell bottle. Observe the cells under the microscope daily. When a large number of vacuoles appear in the cells and they begin to rupture, add FBS to the original medium to increase the concentration to 10%, and then collect the supernatant and centrifuge it at 2000 rpm at 4 °C for 10 min. Filter the supernatant using a 0.45 µm syringe filter, followed by a 0.2 µm syringe filter for the second filtration. Aliquot the filtered supernatant into 1.5 mL cryovials to constitute the viral suspension, and store it at −80 °C for future use.

### 2.5. Virus Infection

Because Aag2 cells are easily suspended, the traditional cell infection procedure was modified. The DENV2 suspension was diluted to the appropriate ratio based on the DENV2 titer to achieve a multiplicity of infection (MOI) of 0.1. This suspension was then directly added to the 12-well plate, and the cells were cultured further. Antibiotics were excluded from the entire procedure to prevent interference with the transfection process, and all steps were performed gently to avoid dislodging the Aag2 cells.

The experimental design included six groups, each with four biological replicates: the no treatment group (N, only Aag2 cells); the EGFP group (E, transfected with the control EGFP plasmid); the gene group (G, transfected with the experimental plasmid); the DENV2 group (D2, DENV2 infection of the N group); the EGFP + DENV2 group (ED2, DENV2 infection of the E group); and the gene + DENV2 group (GD2, DENV2 infection of the G group).

### 2.6. RT-qPCR Detection of Gene Expression

Samples were collected at 2-, 4-, and 6-days post-infection (dpi). Cell density was performed using the Bio-Rad TC20 Automatic Cell Counter (Bio-Rad, Hercules, CA, USA). The cells were then centrifuged at 2000 rpm for 5 min, and the supernatant was transferred to a new EP tube for virus detection. Cell samples were stored in Trizol, followed by RNA extraction for gene expression analysis. To ensure no residual genomic or plasmid DNA carried over into the RT-qPCR reaction, DNase treatment was applied to the extracted RNA. RT-qPCR detection was performed using TransScript^®^ Green One-Step RT-qPCR Super Mix (Transgene, Beijing, China). The reaction conditions were as follows: 45 °C for 5 min; 94 °C for 30 s; 40 cycles of 94 °C for 5 s; and 60 °C for 30 s, followed by a dissociation stage. The primers used in RT-qPCR detection are listed in Table 2. Ribosomal protein S6 (RPS6, LOC5563590) was used as the reference gene, and relative expression levels were calculated using the 2^−ΔΔCT^ method. The EGFP-transfected group was used as the control, and the data were expressed as fold change (FC) [33].

### 2.7. Detection of DENV2 RNA Copies in the Supernatant of Cells After Transfection

DENV2 RNA copies in the cell supernatant were detected using the DENV2 nucleic acid extraction-free detection kit (Suneye Biotechnology, Beijing, China). The reaction system consisted of the following components: 10 μL of 2× DENV2 amplification reaction solution; 1 μL of DENV2 primer and probe mixture; 0.5 μL of RT-qPCR enzyme mixture; 4 μL of cell supernatant; and nuclease-free water to a final volume of 20 μL. The reaction conditions were 50 °C for 10 min, 95 °C for 10 min, followed by 40 cycles of 95 °C for 15 s and 60 °C for 30 s.

### 2.8. Thoracic Microinjection of Adult Female Mosquitoes

Based on the results of DENV2 RNA copies in the supernatant of cells after overexpression, select the target gene and synthesize siRNA for the target gene. Establish a gene interference model in mosquitoes to further study the impact of the gene on DENV2 replication in individual mosquitoes. The siRNAs of these genes were designed and synthesized by Sangon Biotech (Shanghai, China) Co., Ltd. The siRNA sequences are listed in Table 3.

First, prepare the injection reagent. When establishing a mosquito gene interference model, simply mix the siRNA dry powder with PBS buffer at a certain ratio to achieve a siRNA concentration of 80 μM, and then proceed to test the interference effect. When further investigating the effect of genes on DENV2 replication in mosquitoes, siRNA dry powder needs to be diluted in PBS buffer, then mixed with the DENV2 suspension at a titer of 2.5 × 10^8^ PFU/mL to achieve a final siRNA concentration of 80 μM and a viral titer of 5 × 10^7^ PFU/mL. The siRNA–virus mixture was then prepared. Next, a borosilicate capillary glass tube (SUTTER, BF100-50-10) was pulled using a needle puller (SUTTER, MODEL P-1000) to create a microinjection needle. The injection reagent was loaded using a microsample loading gun (Eppendorf, 20 μL), and the needle was mounted on a microinjection pump (Eppendorf, FemtoJet 4i). After 3 to 5 days post-eclosion, female mosquitoes were anesthetized with cold by placing them on a metal bath set to 4 °C. Mosquitoes were injected one by one under a microscope, with an injection volume of 300 nL per mosquito. Mosquitoes injected with target gene siRNA were designated as the treatment group, while those injected with NC siRNA served as the control group. After interference, samples were collected for two consecutive days, with 15 whole mosquitoes collected from each group each day, and RNA was extracted for subsequent analysis.

### 2.9. Quantification of Target Gene Expression in Mosquitoes After siRNA Injection

Using the Trizol method to extract mosquito RNA. The expression levels of target genes in mosquitoes were detected using the HiScript II One Step RT-qPCR SYBR Green Kit. The reaction system consisted of the following components: 10 μL of 2× One-Step SYBR Green Mix, 1 μL of One Step SYBR Green Enzyme Mix, 0.4 μL of Forward Primer (10 μM), 0.4 μL of Reverse Primer (10 μM), 0.4 μL of ROX Reference Dye 1 (50×), 2 μL of RNA template (RNA concentration approximately 150 ng/μL), and double-distilled water to a final volume of 20 μL. The reaction conditions were as follows: 50 °C for 3 min, 95 °C for 30 s, followed by 40 cycles of 95 °C for 10 s and 60 °C for 30 s, with a dissociation stage. Primer sequences are provided in Table 3.

### 2.10. Detection of DENV2 RNA Copies in Mosquitoes After siRNA Injection

The DENV2 RNA copies were detected following mosquito RNA extraction using the GoTaq^®^ Probe 1-Step System Assay Kit (Promega, A6120). The reaction format was as follows: 10 μL of 2× GoTaq^®^ qPCR Master Mix, 0.4 μL of Go Script™ RT Mix for 1-Step RT-qPCR (50X), 1 μL of Forward primer (5′-AAGGACTAGAGGTTAGAGGAGACCC-3′), 1 μL of Reverse primer (5′-CGTTCTGTGCCTGGAATGATG-3′), 1 μL of Hydrolysis probe (FAM-AACAGCATATTGACGCTGGGAGAGACCAGA-BHQ1), 2 μL of RNA template (RNA concentration approximately 150 ng/μL), and double-distilled water to a final volume of 20 μL. The reaction conditions were as follows: 45 °C for 15 min; 95 °C for 2 min; followed by 40 cycles of 95 °C for 15 s and 60 °C for 1 min. The reporter used was FAM, with no quencher.

### 2.11. Statistical Method

All data are presented as the means ± standard deviations (SDs). To compare mRNA expression levels, cell density, and viral copies between two groups, the Shapiro–Wilk test was first used to assess data normality, followed by the Brown–Forsythe test to evaluate the homogeneity of variances. If the data were normally distributed and variances were homogeneous, an unpaired *t*-test was performed. If variances were unequal, a Welch-corrected unpaired *t*-test was applied. For non-normally distributed data, the non-parametric Mann–Whitney test was used. A *p*-value of <0.05 was considered statistically significant. Data processing was performed using Microsoft Office Home and Student 2019, statistical analysis was carried out with IBM SPSS Statistics V27 software, and GraphPad Prism 8.4.2 was used for plotting.

## 3. Results

### 3.1. Constructed an Instantaneous Transfection and Overexpression Model of Aag2 Cells

In this study, based on the transcriptome analysis of the midgut and salivary glands of *Aedes aegypti* after infection with DENV2 (BioProject ID: PRJNA1197782) [30], as well as the transcriptome analysis of Aag2 cells infected with DENV2 [31], 24 genes potentially related to the regulation of *Aedes aegypti* infection by DENV2 were selected. To verify the role of DEGs in the replication of DENV2, a transient transfection and overexpression model of *Aedes aegypti* Aag2 cells was established.

**Figure 2:**
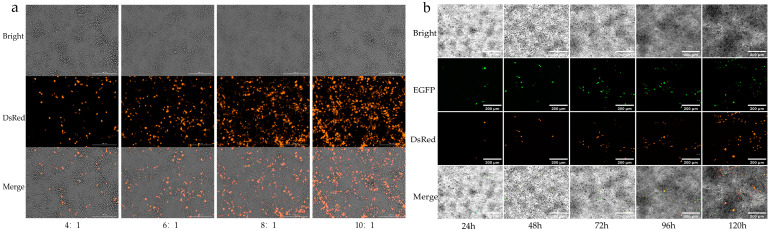
Schematic of fluorescence detection: (**a**) Schematic of fluorescence detection for the optimized transfection ratio. DsRed represents the Red Fluorescent Protein on the plasmid. Observing red fluorescence indicates that the plasmid has been successfully transfected into the cells; (**b**) Schematic of fluorescence detection for control plasmid overexpression. EGFP represents the Enhanced Green Fluorescent Protein, and DsRed represents the Red Fluorescent Protein on the plasmid. Observing red and green fluorescence indicates that the plasmid has been successfully transfected into the cells.

Firstly, to determine the optimal transfection efficiency, a gradient of four transfection reagent-to-plasmid vector ratios (μL:μg) was tested: 4:1, 6:1, 8:1, and 10:1. Fluorescence analysis (Figure 2a) showed that transfection efficiency increased with the volume of transfection reagent. Although the 8:1 and 10:1 ratios yielded similar results, the 8:1 ratio was chosen as the optimal condition for subsequent experiments, as it minimized cytotoxic effects on the cells.

The experimental and control plasmids were overexpressed in Aag2 cells using transient transfection with an 8:1 transfection ratio to establish the overexpression model. To assess the success of plasmid transfection, fluorescence microscopy was used to observe the transfection effect of the control plasmid at 24 h intervals post-transfection. The results (Figure 2b) indicated that, although the transfection efficiency was slightly lower than the previous vector—PSL1180polyUBdsRED (Figure 2a), a clear transfection effect was still observed. To verify successful target gene overexpression, cell samples were collected at 2, 4, and 6 dpi for gene quantification. RPS6 was used as the internal reference gene, and relative gene expression levels were calculated using the fold change (log_10_FC). The results (Table 4) showed that, at 2, 4, and 6 dpi, the log_10_FC values of the G/E group and the GD2/ED2 group were all greater than zero, indicating successful overexpression of the target gene in Aag2 cells. (Where E denotes the EGFP group, transfected with the control EGFP plasmid; G denotes the gene group, transfected with the experimental plasmid; ED2 denotes the EGFP + DENV2 group, i.e., the DENV2-infected group E; and ED2 denotes the gene + DENV2 group, i.e., the DENV2-infected group G.)

### 3.2. The Change in DENV2 RNA Copies in Aag2 Cell Supernatants After Gene Overexpression

To assess the impact of target gene overexpression on viral replication, cells were transfected with the target gene for 24 h, followed by DENV2 infection. The DENV2 RNA copies in Aag2 cell supernatant were quantified at 2, 4, and 6 dpi. Statistical analysis revealed significant differences in DENV2 RNA copies between the GD2 and ED2 groups (*p* < 0.05) (Table 5). Specifically, 19 genes exhibited significant alterations in DENV2 RNA copies. Of these, six genes showed an increase in DENV2 RNA copies, with fold changes (FC) ranging from 1.083 to 1.574; nine genes demonstrated a decrease in DENV2 RNA copies, with FC values ranging from 0.657 to 0.919; and four genes exhibited an initial increase followed by a decrease in RNA copies, with FC values ranging from 0.551 to 1.244.

**Figure 3:**
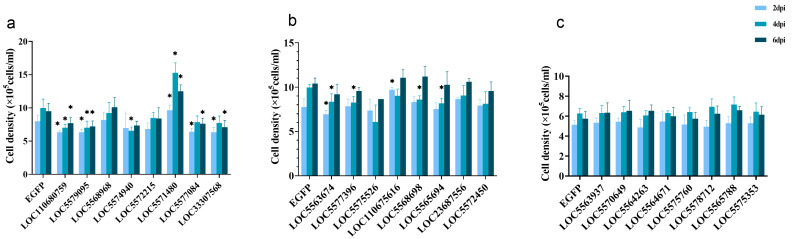
Aag2 cell density: (**a**) Cell density of the first experiment; (**b**) Cell density of the second experiment; (**c**) Cell density of the third experiment. *p* values are indicated as follows: *p* < 0.05 (∗).

To avoid the impact of changes in cell growth rate on viral replication due to gene overexpression, DENV2 infection was conducted 24 h after overexpression of the target gene. Cell density was assessed at 2, 4, and 6 dpi. The 24 genes were divided into three experiments (Figure 3a–c). Statistical analysis comparing the GD2 group to the ED2 group revealed that the cell density of the LOC110680759, LOC5579095, LOC5574940, LOC5577084, LOC33307568, LOC5563674, LOC5577396, LOC5568698, and LOC5565694 groups decreased significantly, while the cell density of the LOC5571480 and LOC110675616 groups increased significantly. No statistically significant differences in cell density were observed for the remaining genes after overexpression.

After overexpression of the target gene in Aag2 cells, cell growth density may be affected, thereby influencing viral copy numbers. To eliminate the potential confounding effect of cell density, this study analyzed both cell density and viral copy number results together. Overexpression of LOC110680759, LOC5579095, LOC5574940, LOC5577084, LOC33307568, and LOC5563674 in cells resulted in a decrease in cell density but an increase in viral copies, indicating that the increase in viral replication is not attributable to changes in cell density. In contrast, overexpression of LOC5568698 led to a decrease in both cell density and viral copies, suggesting that the reduction in viral copies may be due to the diminished cell density, which in turn limited viral replication capacity. For LOC5565694, a decrease in cell density was observed at 4 dpi, although the change in viral copies was not statistically significant. Overexpression of LOC110675616 resulted in an increase in cell density, but no statistically significant change in viral copies was detected. LOC5571480 showed an initial increase followed by a decrease in viral copies, despite an overall increase in cell density. For the remaining genes, no significant differences in cell density were observed, though changes in viral copies were statistically significant. In summary, the overexpression of most genes did not influence viral replication through alterations in cell density, indicating that the statistically significant difference in viral copies in the cell supernatant after overexpression of the target gene in Aag2 cells is valid.

### 3.3. Establishment of a Gene Interference Model in Mosquitoes

**Figure 4:**
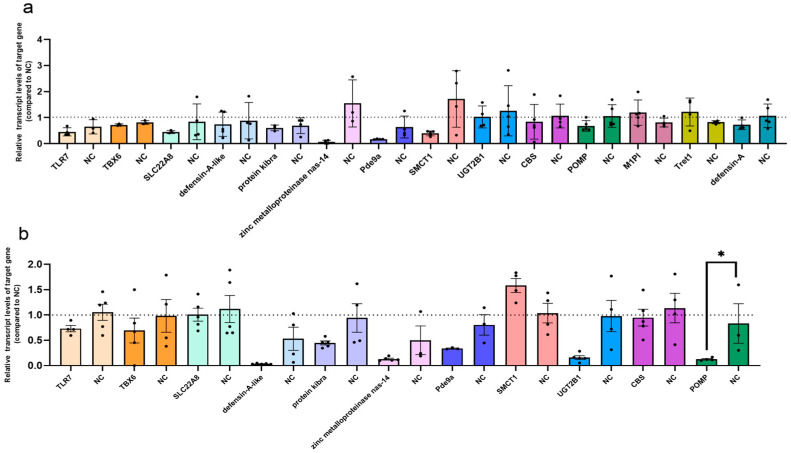
Assessment of the knockdown efficiency of genes in *Aedes aegypti*: (**a**) The knockdown effect of 14 genes was assessed on day 1 post-interference. (**b**) The knockdown effect of 12 genes was assessed on day 1 post-interference. Columns of the same color indicate the experimental group, and the other, the negative control group. NC denotes the negative control. *p* values are indicated as follows: *p* < 0.05 (∗).

To further investigate the effect of DEGs on DENV2 replication, eight genes that led to an increase in DENV2 RNA copies and six genes that resulted in a decrease in DENV2 RNA copies after overexpression were selected from the viral copy data (Table 5). These 14 genes were identified as potentially influential in viral replication. siRNA targeting each gene was designed (Table 3), and mosquito thoracic microinjection was used to introduce the siRNA into *Aedes aegypti* to inhibit target gene expression and establish a gene interference model. The results showed that, on days 1 and 2 post-interference, UGT2B1 was successfully knocked down, with statistically significant differences observed. Additionally, the expression levels of defensin-A, defensin-A-like, POMP, ND4, and SMCT1 were significantly reduced (Figure 4). Based on the changes in DENV2 RNA copies following target gene overexpression (Table 5), six genes—UGT2B1, defensin-A, defensin-A-like, POMP, ND4, and SMCT1—were selected. Subsequently, the siRNA of these six genes was mixed with DENV2 suspension and injected into *Aedes aegypti* through thoracic microinjection to further investigate their roles in DENV2 replication within the mosquito.

### 3.4. Changes in Target Gene Expression and DENV2 RNA Copies in Mosquitoes After siRNA Interference

Based on the changes in DENV2 RNA copies after the overexpression of target genes (Table 5) and the results from the mosquito interference model (Figure 4), the six genes UGT2B1, defensin-A, defensin-A-like, POMP, ND4, and SMCT1 were selected for further functional studies. The siRNAs targeting the six genes were mixed with DENV2 suspension and injected into *Aedes aegypti* through thoracic microinjection. Since the interference effect on day 3 post-injection was less effective than on the first two days, gene expression and viral copies were measured on days 1 and 2 post-injection using RT-qPCR.

**Figure 5:**
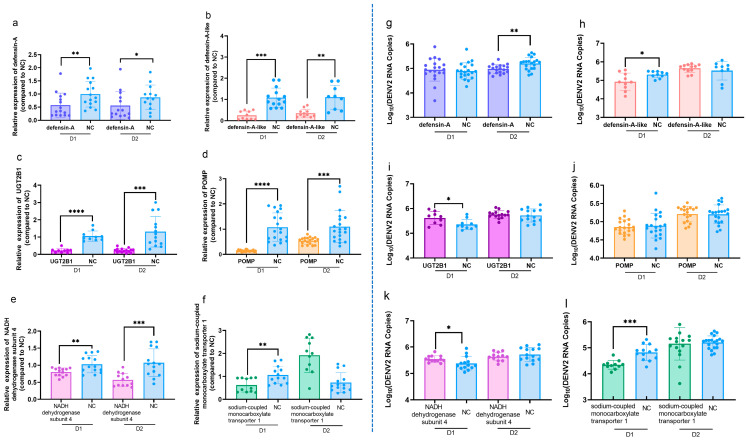
Assessment of the knockdown efficiency of genes (**left**) and comparison of DENV2 RNA copies (**right**) in *Aedes aegypti*: (**a**–**f**) Knockdown efficiency of six genes associated with DENV2 infection and replication in *Aedes aegypti* (**left**); (**g**–**l**) DENV2 RNA copies in *Aedes aegypti* before and after six genes knockdown (**right**). *p* values are indicated as follows: *p* < 0.05 (∗), *p* < 0.01 (∗∗), *p* < 0.001 (∗∗∗), *p* < 0.0001 (∗∗∗∗).

First, RNA was extracted from mosquitoes injected with either target gene siRNA or NC siRNA, and the relative expression levels of the target genes were quantified using RT-qPCR. The results showed that, within 1–2 days post-injection, the expression of defensin-A, defensin-A-like, UGT2B1, POMP, ND4, and SMCT1 was significantly reduced, with statistically significant differences (Figure 5a–f).

Within 1–2 days post-injection, the DENV2 RNA copies in mosquitoes were detected by RT-qPCR, with results shown in Figure 5g–l. Knockdown of defensin-A, defensin-A-like, and SMCT1 led to a reduction in DENV2 RNA copies compared to the control group, with statistically significant differences (Figure 5g,h,l). Knockdown of UGT2B1 and ND4 resulted in an increase in DENV2 RNA copies compared to the control group, with statistically significant differences (Figure 5i,k). Knockdown of POMP did not result in a statistically significant difference in DENV2 RNA copies (Figure 5j).

## 4. Discussion

This study developed a gene overexpression model in Aag2 cells and employed thoracic microinjection techniques in female *Aedes aegypti* to knock down target genes, further investigating the replication roles of DENV2 susceptibility-related genes in *Aedes aegypti*. By conducting research at both the cellular and mosquito levels, genes that directly influence the replication of mosquito-borne viruses and modulate viral loads were identified, suggesting that these genes play a role in the regulatory mechanisms underlying mosquito-borne virus susceptibility.

This study integrates the results of viral copy measurements before and after gene overexpression in Aag2 cells with those from gene knockdown experiments in mosquitoes to facilitate the following discussion.

First, the overexpression of defensin family genes, including defensin-A and defensin-A-like, in Aag2 cells significantly increased DENV2 RNA copies, suggesting their involvement in DENV2 viral replication. Conversely, the knockdown of defensin-A and defensin-A-like in mosquitoes significantly reduced DENV2 RNA copies, further supporting their role in viral replication. These findings suggest that defensin-A and defensin-A-like may play important roles in the viral replication process. Previous research on Drosophila melanogaster provides insights into the innate immune responses of insects. Insects’ first line of defense against pathogens involves both cellular mechanisms and a series of antimicrobial peptides (AMPs), such as defensins and cecropins [34]. AMPs are key components of humoral immunity [35]. In Drosophila melanogaster, seven distinct AMP families have been identified, each showing varied specificity toward different microorganisms [36]. However, the AMPs in mosquitoes differ notably from those in fruit flies, with defensins and cecropins being predominant in mosquitoes [37]. In *Aedes aegypti*, defensins primarily target Gram-positive bacteria [38]. Recent studies, however, have highlighted that the defensin gene family also plays a role in the immune response to Chikungunya and Zika viruses in *Aedes aegypti* [39]. In contrast, our findings show that overexpression of defensin-A and defensin-A-like increases viral copies in Aag2 cells, while knockdown reduces viral copies in mosquitoes. This suggests that the defensin protein family may exhibit distinct roles in DENV2 replication.

Secondly, the overexpression of UGT2B1 in Aag2 cells resulted in a significant downregulation of DENV2 RNA copies, while knockdown of UGT2B1 in mosquitoes led to an increase in DENV2 RNA copies. Studies have shown that UGT2B1, along with esterases, is one of the most important non-P450 enzymes, playing a significant role in drug metabolism [40]. UGT is a major family of drug-metabolizing enzymes involved in glucuronidation and the subsequent elimination of drugs and small lipophilic molecules [41], making it one of the primary enzymes in drug metabolism. Glucuronidation serves as a major detoxification pathway for both endogenous and exogenous compounds and is increasingly recognized for its role in drug clearance and elimination [42].

Additionally, the overexpression of cytochrome P450 4c21 (CYP4c21) significantly downregulated DENV2 RNA copies. Cytochrome P450 enzymes are membrane-bound hemoproteins involved in the detoxification of xenobiotics, as well as cellular metabolism and homeostasis [43]. Significant progress has been made in understanding resistance mechanisms in mosquitoes based on cytochrome P450 [44]. Traditionally, it was believed that drug oxidation and conjugation mediated by UGT and P450 occurred independently. However, recent studies have shown functional cooperation between UGT and P450, facilitating drug metabolism. Increasing evidence suggests that interactions occur between UGT subtypes or between P450 and UGT, with UGT function being altered due to protein–protein interactions [45,46]. Based on the results of this study, it is hypothesized that both UGT and P450 enzymes may play roles in the metabolic processes during DENV2 replication in *Aedes aegypti*. However, their specific mechanisms and interactions warrant further investigation.

The overexpression of mitochondrial NADH dehydrogenase subunit 4 (ND4) in Aag2 cells significantly increased DENV2 RNA copies, while knockdown in mosquitoes resulted in a reduction in DENV2 RNA copies. NADH dehydrogenase is known to consist of subunits encoded by mitochondrial genes [47]. In fruit flies and other insects, the NADH dehydrogenase subunit gene family represents the largest family in the mitochondria. Mitochondrial DNA (mtDNA) has proven to be an effective genetic marker for determining gene flow within species and is frequently employed in population genetic studies. For instance, Paupy et al. [48] used mtDNA-ND4 to investigate genetic variation between *Aedes aegypti* populations from the Cameron Highlands and domesticated populations. However, no research has yet linked this gene to insect–virus interactions.

In this study, overexpression of proteasome maturation protein (POMP) in Aag2 cells significantly inhibited DENV2 replication, but no significant difference in DENV2 replication was observed in mosquitoes after knockdown. As a molecular chaperone, POMP is essential for the assembly of both standard proteasomes and immunoproteasomes. It mediates the connection between the 20S proteasome and the endoplasmic reticulum, facilitating key steps in the formation of the 20S core complex at the ER [49]. The role of POMP in the infection process of *Aedes aegypti* by DENV2 requires further investigation.

Studies have also identified two sodium-coupled monocarboxylate transporters (SMCT1 and SMCT2), which mediate the active transport of monocarboxylates, such as lactate and ketone bodies [50]. These metabolites play a crucial role in energy metabolism, serving not only as waste products of glucose or fatty acid metabolism but also as important energy sources [51,52,53]. SMCT1, a high-affinity transport protein, has been reported to be expressed in various tissues, including the colon, small intestine, kidneys, thyroid, and brain [54,55]. In this study, overexpression of SMCT1 in Aag2 cells significantly inhibited DENV2 replication, and knockdown of this gene in mosquitoes also reduced DENV2 replication. These results suggest that SMCT1 may play an important role in DENV2 replication in mosquitoes, although further investigation is required to confirm this hypothesis.

In summary, the findings of this study suggest that genes such as defensin-A, defensin-A-like, UGT2B1, cytochrome P450, and SMCT1 play a role in the process of DENV2 replication in *Aedes aegypti*. However, the precise mechanisms underlying their involvement require further investigation.

## 5. Conclusions

This study combines previous transcriptome data from the midgut and salivary glands of *Aedes aegypti* infected with DENV2 (BioProject ID: PRJNA1197782) [30], as well as transcriptome data from Aag2 cells of *Aedes aegypti* infected with DENV2 [31], to selected 24 genes, and further conducts functional analysis of these genes in Aag2 cells and *Aedes aegypti* Tables S1 and S2. The results revealed that the overexpression of genes such as defensin-A, UGT2B1, SMCT1, POMP, CYP4c21, M1Pi, TLR7, TBX6, CBS, Tret1, SLCO2A1, SLC22A8, GNBPB6, Kibra, and Pde9a in Aag2 cells also influenced virus replication in the mosquito vector. Similarly, knockdown of defensin-A, defensin-A-like, SMCT1, UGT2B1, and ND4 in mosquitoes affected viral replication and altered viral copies. These findings suggest that the genes listed above are involved in the infection and replication processes of DENV2. They represent potential target genes for further investigation into viral infection in mosquitoes. Additionally, this study provides valuable insights for targeting the regulation of mosquito-borne viral infections and transmission, offering a foundation for exploring the complex mechanisms of mosquito–pathogen interactions.

## Figures and Tables

**Table 1:** Information on the 24 DENV2-infected DEGs for further overexpressed.

No.	Vectorbase ID	Gene ID	Gene Description	Mosquito Salivary Glands		Mosquito Midgut		Aag2 Cell	
				log_2_FC	padj	log_2_FC	padj	log_2_FC	padj
1	AAEL003841	LOC110680759	defensin-A	0.402	0.871	2.992	0.003	-	-
2	AAEL003857	LOC5579095	defensin-A-like	1.420	0.007	2.348	0.000	-	-
3	AAEL007268	LOC5568968	protein kibra	2.479	0.013	3.299	0.000	-	-
4	AAEL011559	LOC5574940	zinc metalloproteinase nas-14	3.951	0.030	5.969	0.000	-	-
5	AAEL009630	LOC5572215	Pde9a/high affinity cGMP-specific 3′,5′-cyclic phosphodiesterase 9A	2.276	0.000	2.485	0.000	-	-
6	AAEL021099	LOC5571480	SLCO2A1/solute carrier organic anion transporter family member 2A1	0.700	0.858	2.501	0.004	-	-
7	AAEL012981	LOC5577084	SLC22A8/solute carrier family 22 member 8	1.516	0.641	2.442	0.003	-	-
8	AAEL018680	LOC33307568	ND4/NADH dehydrogenase subunit 4	1.137	0.086	2.006	0.021	-	-
9	AAEL000556	LOC5563674	perlucin	7.310	0.045	−1.221	1.000	-	-
10	AAEL003163	LOC5577396	protein fork head	3.424	0.000	3.982	0.008	-	-
11	AAEL011900	LOC5575526	beta-1,4-glucuronyltransferase	2.821	0.002	2.217	0.001	-	-
12	AAEL023898	LOC110675616	mucin-5AC-like	2.295	0.001	2.458	0.000	-	-
13	AAEL007064	LOC5568698	GNBPB6/beta-1,3-glucan-binding protein	4.059	0.015	1.358	0.542	-	-
14	AAEL004923	LOC5565694	neuronal acetylcholine receptor subunit beta-3	2.499	0.000	−0.455	1.000	-	-
15	AAEL017136	LOC23687556	CYP4c21/cytochrome P450 4c21	2.034	0.001	3.404	1.000	-	-
16	AAEL001835	LOC5572450	SMCT1/sodium-coupled monocarboxylate transporter 1	1.032	0.686	2.544	0.000	-	-
17	AAEL014246	LOC5563937	UGT2B1/UDP-glucuronosyltransferase 2B1	-	-	-	-	2.164	0.000
18	AAEL008467	LOC5570649	CBS/cystathionine beta-synthase	-	-	-	-	2.103	0.000
19	AAEL004174	LOC5564263	TBX6 T-box transcription factor	-	-	-	-	1.690	0.000
20	AAEL014555	LOC5564671	POMP proteasome maturation protein	-	-	-	-	1.389	0.009
21	AAEL002721	LOC5575760	PEM/protein extra macrochaetae	-	-	-	-	1.253	0.000
22	AAEL013828	LOC5578712	M1PI/ethylthioribose-1-phosphate isomerase	-	-	-	-	1.198	0.000
23	AAEL014972	LOC5565788	Tret1/facilitated trehalose transporter Tret1	-	-	-	-	1.182	0.000
24	AAEL002583	LOC5575353	TLR7 toll-like receptor 7	-	-	-	-	1.175	0.000

Note: VectorBase ID is the number used to identify a gene in the VectorBase database; Gene ID originates from NCBI’s Entrez Gene database, which is the unique numerical identifier assigned by NCBI for each gene. In the adjusted *p*-value column, a value of 0.000 indicates *p* < 0.001, and the difference is statistically significant.

**Table 2:** Primers for the 24 overexpressed genes related to DENV2 infection and replication.

No.	Gene ID	Forward Primer (5′ to 3′)	Reverse Primer (5′ to 3′)	Length (bp)
1	LOC110680759	CGCACTTTACGCTTTCGAG	AGAGCCAGGAAACAAATGACA	138
2	LOC5579095	GAAGCTCGCCCTTTTGCC	CACAAGCACTATCACCAACGC	136
3	LOC5568968	TGATCATATTAACAAGAAGACCAC	CGATCTGAGGGTCATAGC	128
4	LOC5574940	CCACGCCAAACTGATTCGAGA	AAGACCTCGATTACGACCT	148
5	LOC5572215	CCGGTGTAATACTGAATCAACCA	TACGTCCAAGCTGTAGGCTC	116
6	LOC5571480	TCGACGAAAAGGATAAAACCG	TTTTACCCCACACCAAGCA	126
7	LOC5577084	TTGATCAACGTTTTCGAGCTG	TCATTATAACGCCTCCCGCTGT	91
8	LOC33307568	AGGCTTTAATTGCTTATTCTTCG	AGCCAAACAAAATAACCCAG	143
9	LOC5563674	TGTGGTCAACAGCGAAGCAA	CGGTTTTCCATTGGCGAT	148
10	LOC5577396	TTCTGGACTTTGCATCCCGAC	TCCGGACTCGTTGCATCCAT	146
11	LOC5575526	TACCAGAGAGTCATTTAGTTCGTC	CTCGACACGTTGAAGTACGG	148
12	LOC110675616	TGAATCGAAACCCAATCGGACA	TCGGAAGTCCAACGAAGCAC	148
13	LOC5568698	GTCACGTGGCAATCGAAACC	CACATTCGGGTTGTCTCCGA	94
14	LOC5565694	ATATTGACGGTTTTCATCAAGCA	GGCTCAACTTCCAAACCAGT	146
15	LOC23687556	TGGATATCAACAATAATCCGAA	TTCCGATAATCGCTGGTCA	136
16	LOC5572450	CATGACTGTGATCCCGTCT	CACAAACAATCCCGGTAGGC	111
17	LOC5563937	ACTTCCCGAACATCACCGAGACT	CCACCACCAGAGCCACCATATCTA	249
18	LOC5570649	CGGAGAAGATGTCCAACGAGAAGG	CGCCAAAGGATTGCCCGAGTT	185
19	LOC5564263	GGACGAAAACTACTGCGTGC	GGTACATCCGTTGGGGACTC	122
20	LOC5564671	CGGAACTGAACTACGAACAACACC	TGGACGGCAGGAACGGCATA	141
21	LOC5575760	GTTCATGCCGAAGAACCGC	AGGCCTGAACGGGATCAAAA	128
22	LOC5578712	GCAATACTGGATCACTGGCGACTG	CTCCAACAACTACTGCCGCTACTC	237
23	LOC5565788	GAGGTACGAGGAACACTTGGACTG	AGGAACATCAGCAACAGGAATGGT	149
24	LOC5575353	CCGTACCGAGGCAACAACTATACC	GCTGCGGAAGCTCCACCATT	190
25	RPS6	CGTCGTCAGGAACGTATCC	TTCTTGGCAGCCTTAGCAG	119

**Table 3:** siRNA sequence.

No.	Gene ID	Sense Strand (5′-3′)	Antisense Strand (5′-3′)
1	LOC33307568	GCUACUUGUUUAUUUAUAATT	UUAUAAAUAAACAAGUAGCTT
2	LOC110680759	CGAUUAUCACAUCAUUCAATT	UUGAAUGAUGUGAUAAUCGTT
3	LOC5564671	CGGUCAUGUUGCUCCACUATT	UAGUGGAGCAACAUGACCGTT
4	LOC5572450	GACAGACUAUGGUUCAAUATT	UAUUGAACCAUAGUCUGUCTT
5	LOC5579095	GCUACUGCAACUCCAAGAATT	UUCUUGGAGUUGCAGUAGCTT
6	LOC5563937	CGAGUAACGUACUGAUCAATT	UUGAUCAGUACGUUACUCGTT
7	LOC5575353	GAUCCUGUUCGAAAGUCAATT	UUGACUUUCGAACAGGAUCTT
8	LOC5564263	AGUUGAAGAUCGACCACAATT	UUGUGGUCGAUCUUCAACUTT
9	LOC5571480	GCGGAGAUCUCAAGAUCUATT	UAGAUCUUGAGAUCUCCGCTT
10	LOC5574940	GGAUCAUGUUAGACCGGAATT	UUCCGGUCUAACAUGAUCCTT
11	LOC5572215	GGAAGAAAGAGAAGAUUAATT	UUAAUCUUCUCUUUCUUCCTT
12	LOC5570649	CGAAGAAUCUCCAGAUGAATT	UUCAUCUGGAGAUUCUUCGTT
13	LOC5578712	CCAAGUACAUAGAUGUUAATT	UUAACAUCUAUGUACUUGGTT
14	LOC5565788	GGAAGAUGCUGUUGUACAUTT	AUGUACAACAGCAUCUUCCTT
15	NC	UUCUCCGAACGUGUCACGUTT	ACGUGACACGUUCGGAGAATT

Note: NC denotes the negative control.

**Table 4:** The relative expression levels of 24 genes (log_10_FC).

No.	Gene ID	2 dpi		4 dpi		6 dpi	
		G/E	GD2/ED2	G/E	GD2/ED2	G/E	GD2/ED2
1	LOC110680759	6.27	5.92	4.72	5.25	4.70	4.04
2	LOC5579095	4.01	3.81	3.70	3.68	5.68	2.79
3	LOC5568968	3.49	3.49	2.41	2.31	2.21	2.11
4	LOC5574940	4.43	4.41	4.87	3.77	5.17	2.63
5	LOC5572215	4.62	4.53	4.35	3.27	5.64	2.80
6	LOC5571480	4.31	4.04	4.79	3.40	5.11	2.85
7	LOC5577084	4.44	4.64	4.46	3.68	4.81	3.25
8	LOC33307568	2.61	2.55	1.21	1.28	1.65	0.97
9	LOC5563674	4.05	3.59	3.16	3.33	3.14	2.46
10	LOC5577396	2.14	1.29	2.10	1.07	1.19	0.67
11	LOC5575526	3.97	2.03	3.77	4.06	3.17	2.35
12	LOC110675616	3.92	2.63	4.13	3.90	3.67	3.22
13	LOC5568698	5.04	5.59	3.95	3.49	4.60	3.27
14	LOC5565694	4.05	5.54	3.61	3.38	3.94	3.05
15	LOC23687556	5.19	5.10	3.44	3.88	4.41	3.80
16	LOC5572450	3.63	6.32	3.65	2.82	4.74	2.28
17	LOC5563937	1.87	1.89	1.39	1.19	1.03	0.96
18	LOC5570649	1.23	0.99	0.73	0.52	0.29	0.34
19	LOC5564263	3.44	3.05	2.49	2.62	2.85	2.38
20	LOC5564671	2.77	2.75	2.36	2.08	2.00	1.95
21	LOC5575760	2.89	2.81	2.41	2.34	2.38	2.16
22	LOC5578712	2.45	2.38	2.05	2.05	1.88	1.70
23	LOC5565788	3.35	3.30	3.03	2.98	3.31	2.40
24	LOC5575353	4.27	3.88	3.08	3.38	3.66	2.96

Note: G/E represents the relative expression of Group G compared to Group E; GD2/ED2 represents the relative expression of Group GD2 compared to Group ED2.

**Table 5:** Statistical analysis of DENV2 RNA copies after the target gene is overexpressed.

Gene ID	2 dpi		4 dpi		6 dpi	
	*p* Value	FC	*p* Value	FC	*p* Value	FC
LOC110680759	0.053	1.198	0.018	1.428	0.112	1.072
LOC5579095	0.002	1.315	0.012	1.552	0.445	1.032
LOC5568968	0.006	1.204	0.323	1.183	0.007	0.724
LOC5574940	0.007	1.227	0.039	1.445	0.772	1.017
LOC5572215	0.040	1.083	0.028	1.389	0.209	0.916
LOC5571480	0.014	1.244	0.978	0.995	0.001	0.551
LOC5577084	0.000	1.324	0.138	1.368	0.672	0.979
LOC33307568	0.000	1.345	0.005	1.574	0.892	1.006
LOC5563674	0.959	0.999	0.596	0.944	0.303	1.095
LOC5577396	0.801	0.991	0.126	1.140	0.445	1.046
LOC5575526	0.889	1.004	0.524	1.092	-	-
LOC110675616	0.341	0.971	0.265	0.908	0.895	0.991
LOC5568698	0.377	0.977	0.015	0.790	0.109	0.852
LOC5565694	0.902	1.006	0.465	1.042	0.409	1.053
LOC23687556	0.855	0.995	0.006	0.657	0.186	1.045
LOC5572450	0.419	1.043	0.018	0.668	0.541	1.071
LOC5563937	0.813	0.991	0.775	0.986	0.008	0.893
LOC5570649	0.621	1.021	0.672	1.016	0.033	0.884
LOC5564263	0.048	1.126	0.505	1.023	0.001	0.837
LOC5564671	0.260	1.079	0.014	0.887	0.095	0.942
LOC5575760	0.178	1.057	0.086	0.929	0.003	0.915
LOC5578712	0.248	1.059	0.007	0.859	0.019	0.919
LOC5565788	0.175	1.072	0.435	0.967	0.000	0.907
LOC5575353	0.005	1.171	0.814	1.010	0.001	0.880

Note: 0.000 stands for less than 0.001 with a significant difference.

## Data Availability

All data generated or analyzed during this study are included in this published article.

## Supplementary Materials

The following supporting information can be downloaded at: https://www.mdpi.com/article/10.3390/v17010067/s1, Table S1. Differentially expressed genes in salivary glands and midgut of DENV-2-infected Aedes aegypti mosquitoes compared with DENV-2-uninfected Aedes aegypti. Mosquitoes; Table S2. Differentially expressed genes of Aag2 cell line from DENV-2-infected cells compared to uninfected cell.
